# Prognostic Heterogeneity of MRE11 Based on the Location of Primary Colorectal Cancer Is Caused by Activation of Different Immune Signals

**DOI:** 10.3389/fonc.2019.01465

**Published:** 2020-01-17

**Authors:** Chuan-Wen Fan, Maria Kopsida, You-Bin Liu, Hong Zhang, Jing-Fang Gao, Gunnar Arbman, Si-Yu-Wei Cao, Yuan Li, Zong-Guang Zhou, Xiao-Feng Sun

**Affiliations:** ^1^Institute of Digestive Surgery, State Key Laboratory of Biotherapy and Cancer Center, West China Hospital, Sichuan University, and Collaborative Innovation Center for Biotherapy, Chengdu, China; ^2^Department of Oncology, Clinical and Experimental Medicine, Linköping University, Linköping, Sweden; ^3^Department of Medical Sciences, Örebro University, Örebro, Sweden

**Keywords:** colorectal cancer (CRC), left-sided colon and rectal cancer (LSCRC), MRE11, tumor-infiltrating inflammatory cells (TIICs), survival or prognosis

## Abstract

**Background:** MRE11 plays an important role in DNA damage response for the maintenance of genome stability, and is becoming a prognostic marker for cancers, including colorectal cancer (CRC). However, the correlations of MRE11 to prognosis and tumor-infiltrating inflammatory cells (TIICs) in different locations of CRC remains unclear.

**Methods:** Among Swedish and TCGA-COREAD patients, we investigated the association of MRE11 expression, tumor-infiltrating inflammatory cells (TIICs) and microsatellite status with survival in right-sided colon cancer (RSCC) and left-sided colon and rectal cancer (LSCRC). The signaling of MRE11-related was further analyzed using weighted gene co-expression network analysis and ClueGO.

**Results:** High MRE11 expression alone or combination of high MRE11 expression with high TIICs was related to favorable prognosis in LSCRC. Moreover, high MRE11 expression was associated with favorable prognosis in LSCRC with microsatellite stability. The relationships above were adjusted for tumor stage, differentiation, and/or TIICs. However, no such evidence was observed in RSCC. Several signaling pathways involving MRE11 were found to be associated with cell cycle and DNA repair in RSCC and LSCRC, whereas, the activation of the immune response and necrotic cell death were specifically correlated with LSCRC.

**Conclusions:** High MRE11 expression is an independent prognostic marker in LSCRC and enhanced prognostic potency of combining high MRE11 with high TIICs in LSCRC, mainly due to differential immune signaling activated by MRE11 in RSCC and LSCRC, respectively.

## Introduction

Although colorectal cancer (CRC)-related death has decreased over the last decades, it still remains one of the most common leading causes of cancer-related mortality worldwide (1), as a significant proportion of cancer patients develop disease recurrence and metastasis. Various risk factors for CRC have been identified, including familial history, age, primary tumor location, and microsatellite instability (MSI) status (2).

Typically, CRC can be divided into right-sided colon cancer (RSCC) and left-sided colon and rectal cancer (LSCRC) by their primary tumor located in proximal or distal of splenic flexure, due to different embryonic origin. Among many discrepancies in primary tumor from different location (3), differences in their clinical and biological characteristics of RSCC and LSCRC, such as genome-wide hypermethylation via the CpG island methylator phenotype (CIMP), hypermutated state via microsatellite instability and BRAF mutation, indicate that primary tumor location might have a potential impact on the prognosis and treatment efficacy (4, 5). Although primary tumor site has not been accounted by the European Society for Medical Oncology consensus guidelines for the clinical management of patients with metastatic CRC (mCRC), it has been mentioned in both National Comprehensive Cancer Network (NCCN) guidelines and Pan-Asian Adapted ESMO Consensus Guidelines (4, 6, 7), but not explored among the European population. According to several studies, the different origin of tumors consequently leads to differential gene expression, mutation profiles and activation of diverse signaling pathways, and as a result to more or less responsive to specific treatments (8). Such an example is RSCC, which is more responsive to VEGFA inhibitors due to lower frequency of BRAF mutations in RSCC (8). It is, therefore, crucial to advance our knowledge regarding the molecular landscape of colorectal carcinogenesis and discover novel molecular biomarkers with prognostic and predictive information, either involved in DNA damage/repair pathways which are fundamental for maintaining genome stability (9). The MRE11, as a member of DNA damage response (DDR), preserves genome integrity and has recently received attention as such a potential biomarker in CRC, due to its pivotal role in the DNA damage/repair pathways by: (1) sensing DNA double-strand breaks (DSBs) as scans along the DNA via facilitated diffusion to detect free ends and induces the redistribution of the DDR proteins to the damaged site at high concentration, (2) halting cell cycle progression and promoting repair through either non-homologous end joining (NHEJ) or homologous recombination (HR), and (3) governing the activation of the ATM kinase to promote DSB repair and DSB signal amplification (10, 11). DSBs also increase accordingly during the process of cancer cell division, due to genomic instability which reduces cell cycle length and cell growth, respectively. Thus, it was hypothesized that MRE11 expression is potentially higher in CRC compared to normal tissue and this overexpression can be associated with higher chances of genomic instability (12).

Several research groups explored the above hypothesis and indeed MRE11 expression became an indicator for cancer survival, however, its various roles remain controversial in the different types of cancers, or even among different histological types of the same cancer (12–15). As a result, it was of high importance to explore MRE11 expression and cancer survival in relation to other factors that affect the clinicopathological significance in CRC, such as microsatellite instability (MSI) (16). MSI, a genome-wide alteration in repetitive DNA sequence caused by deficiencies in DNA mismatch repair machinery, is an additional genetic pathway involved in CRC carcinogenesis, which is responsible for almost 10–15% of sporadic CRC and almost the total amount of all hereditary non-polyposis CRC (16). In our previous study, MRE11 complex was found to have different clinicopathological significance in CRC with different MSI status, while each component of the complex had an individual role. In parallel, the impact of the tumor microenvironment in CRC was also investigated, emphasizing on the role of inflammatory cell infiltration, which can contribute either positively or negatively in CRC.

Interestingly, according to a recent study, deficient MRE11 predicts better prognosis independent of treatment in the long-term disease-free survival (DFS) and overall survival (OS): an inconsistent result compared to our previous findings, although we did not further analyze the prognostic value of MRE11 in CRC (13, 14). In the meanwhile, still little is known about the heterogeneous roles of MRE11 for prognosis and whether it contributes to the location of primary CRC.

After taking into consideration the differences between RSCC and LSCRC with regard to embryological origin, molecular features, microbiome, and treatment response (3, 4, 15), in this study, we investigated the clinical impact of MRE11 expression in: (1) a cohort of 9 medical centers from Southeast Swedish Health Care Region and (2) the TCGA-COREAD cohort to figure out the MRE11 prognostic significance in RSCC and LSCRC and whether this significance could be location-dependent or not. In addition, given that MRE11 expression is potentially associated with different microsatellite status and tumor-infiltrating inflammatory (TIICs), two genetic markers of high clinical importance and prognostic implications the prognostic correlation of MRE11 expression and TIICs or MSI status was also investigated (16, 17).

## Materials and Methods

### Patients

Cohort 1 was selected for detecting the expression of MRE11 protein. Two hundred seven primary CRC, 39 normal mucosa, and 21 metastatic lymph node samples from the Southeast Swedish Health Care Region, including hospitals in Linköping, Norrköping, Motala, Jönköping, Kalmar, Oskarshamn, Västervik, Eksjö, and Värnamo were analyzed (18, 19). The detailed parameters are summarized in Table S1.

Cohort 2 was selected for detecting the expression of MRE11 mRNA. Level-3 data of RNA-seq and clinicopathology of TCGA colorectal samples (COREAD) were obtained from UCSC Xena (https://xenabrowser.net/hub/). The primary CRC (*N* = 596) and the normal colorectal tissue (*N* = 51) were included, after excluding cases without clinical survival data. The detailed parameters were summarized in Table S2.

### Immunohistochemistry

Immunostaining analysis of MRE11 was performed as described in our previous study (19). We strictly adhered to the standard biosecurity and institutional safety procedures of Linköping University during the performance of the experiments. Details of the immunohistochemistry assay are provided in the Supplementary Methods. Images were captured with Aperio CS2 slide scanner system (Leica Biosystems, Wetzlar, Germany), using a 40x magnification.

### The Evaluation of TIICs

The evaluation of TIICs was followed by a previous study (20). Details of the evaluation of TIICs are provided in the Supplementary Methods.

### Construction of Weighted Gene Co-expression Network

The R package WGNCA was performed for co-expression network constructions (21). In short, the outlier samples were removed for the subsequent analysis (Figure S3A). A power of 3 was selected to calculate topological overlap distance. Next, correlations among gene expression modules and clinical traits including MRE11 expression were determined. Details of WGCNA procedure are provided in the Supplementary Methods.

### ClueGO-CluePedia Functional Analysis

ClueGO and CluePedia were used to investigate MRE11 signaling network (22). Details of ClueGO-CluePedia functional analysis are provided in the Supplementary Methods.

### Statistical Analyses

Data analysis was performed using SPSS 22.0 software package (SPSS Inc.). For differentially expressed gene (DEGs) screening in cohort 2, R package “limma” was applied. Kaplan–Meier curves of OS of the patients in cohort 1 and cohort 2 were generated by GraphPad Prism 6.0. The *P* < 0.05 was considered statistically significant. Details are provided in the Supplementary Methods.

## Results

### MRE11 Expression in CRC as Well as in RSCC and LSCRC

**Figure 1:**
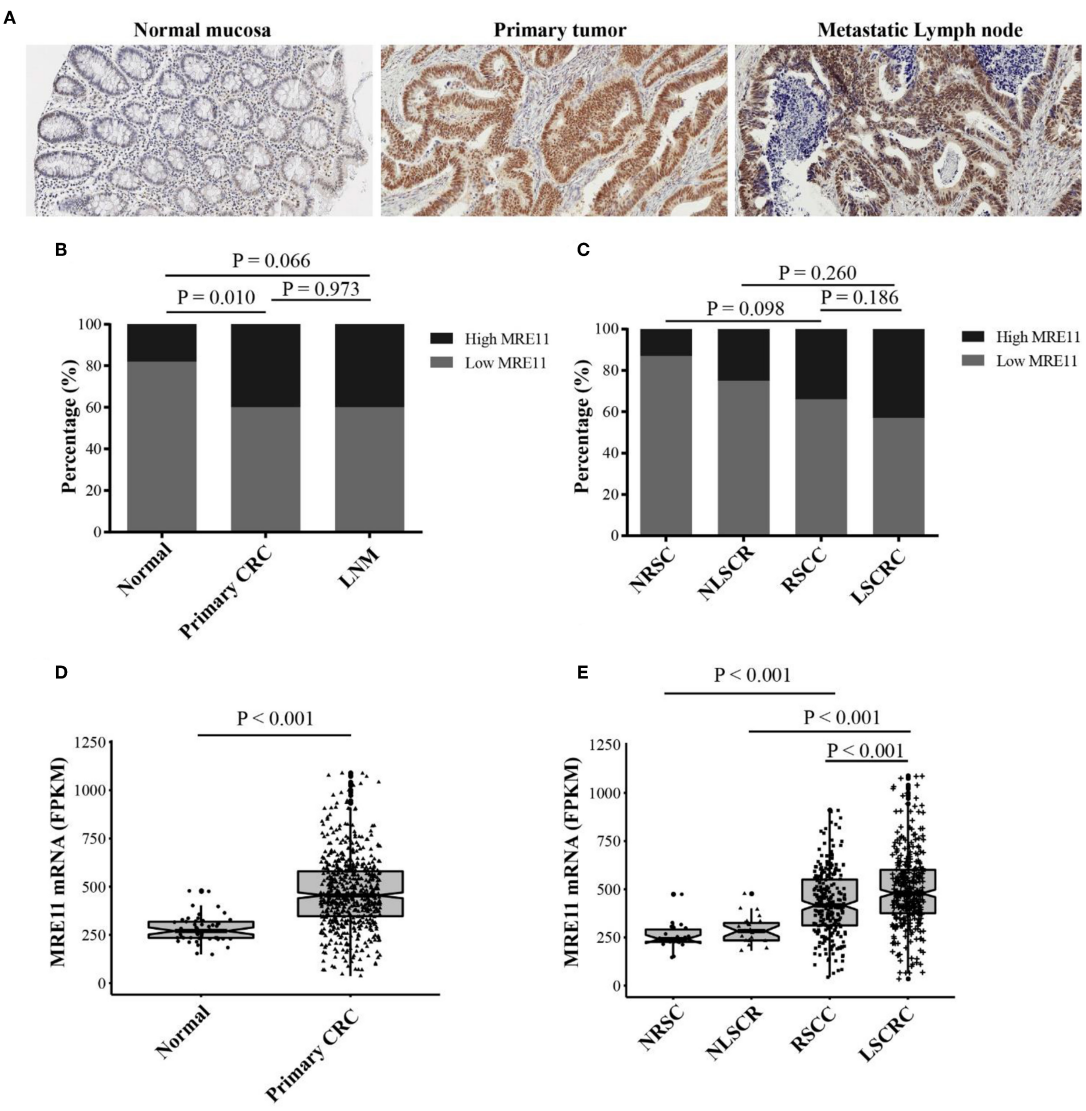
MRE11 expression in CRC. **(A)** Representative staining intensities of MRE11 in normal mucosa, primary CRC, and metastatic lymph node (×20). **(B)** MRE11 expression in normal mucosa, primary CRC tissue, and metastatic lymph node from the cohort 1. **(C)** MRE11 expression in normal right-sided colon mucosa (NRSC), normal left-sided colorectal mucosa (NLSCR), RSCC tissue and LSCRC tissue from the cohort 1. **(D)** MRE11 expression in normal mucosa, primary CRC tissue from the cohort 2. **(E)** MRE11 expression in normal right-sided colon mucosa (NRSC), normal left-sided colorectal mucosa (NLSCR), RSCC tissue and LSCRC tissue from the cohort 2.

MRE11 protein expression was evaluated in normal mucosa, primary tumor and lymph node metastasis (LNM) sections by immunohistochemistry (Figure 1A). MRE11 was highly expressed in primary CRC and metastatic lymph node tissue compared to normal tissue (Figure 1B), although the expression was similar in both tumor and LNM. Furthermore, we analyzed whether MRE11 protein expression was RSCC or LSCRC dependent. A trend of high MRE11 expression in LSCRC and corresponding normal mucosa compared to the expression in RSCC and corresponding normal mucosa was observed, however, the differences did not reach statistical significances (Figure 1C).

We also analyzed the mRNA expression levels of MRE11 based on the cohort 2 that is currently available from TCGA database. The mRNA levels were significantly higher in CRC compared to the normal tissue (Figure 1D). Moreover, mRNA levels of LSCRC and corresponding normal mucosa were increased compared to the mRNA levels of RSCC and corresponding normal mucosa (Figure 1E). Nevertheless, there was no available data for LNM in cohort 2.

### The Prognostic Significance of MRE11 Expression in RSCC and LSCRC

**Figure 2:**
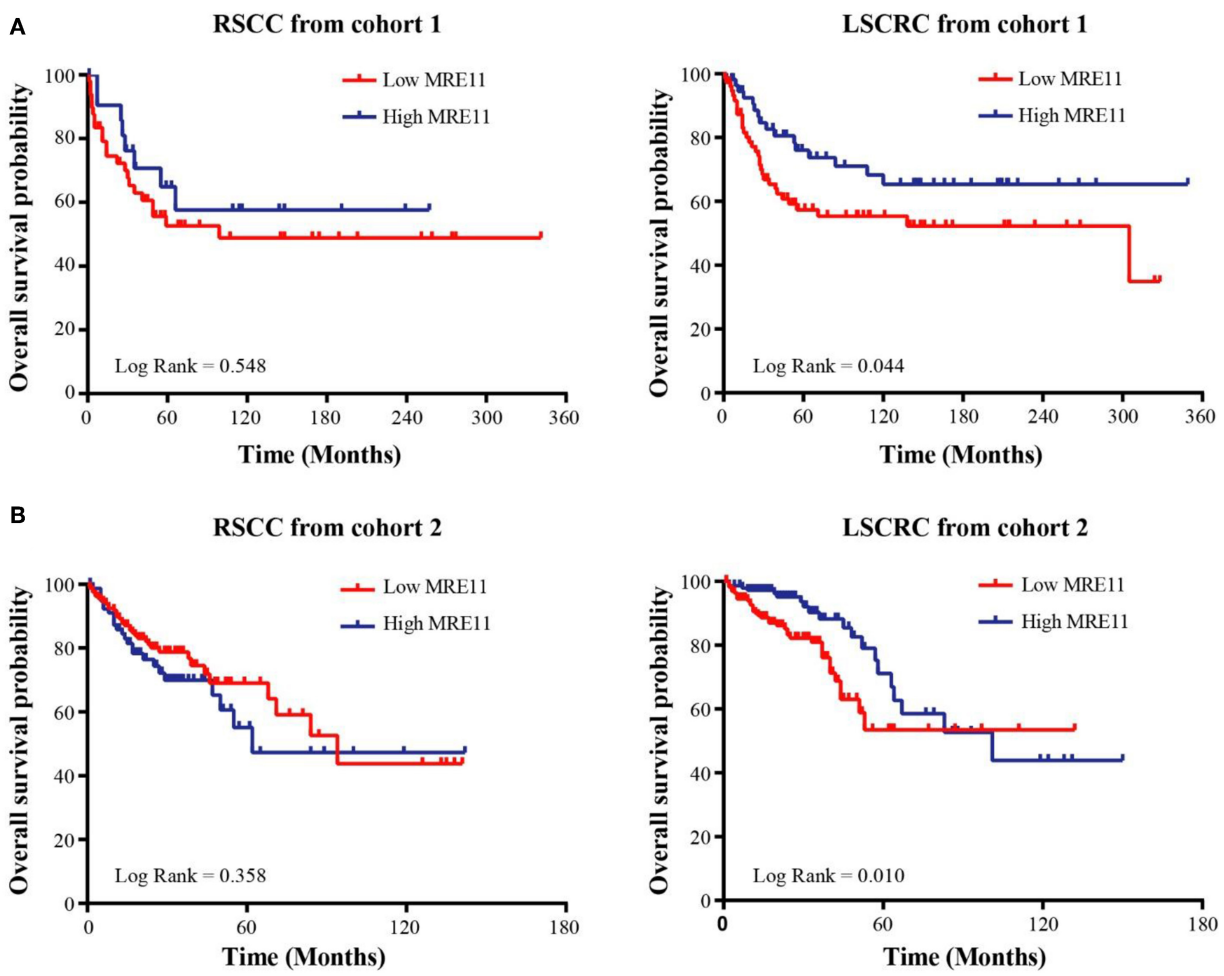
Association of MRE11 expression with prognosis in RSCC and LSCRC. **(A)** Kaplan-Meier OS curves of 74 RSCC patients (left panel) and 132 LSCRC patients (right panel) stratified by MRE11 expression from cohort 1. **(B)** Kaplan-Meier OS curves of 251 RSCC patients (left panel) and 328 LSCRC patients (right panel) stratified by MRE11 mRNA expression from cohort 2.

To explore whether MRE11 expression is related to survival in the patients with CRC or with different location of CRC, the association between MRE11 expression in CRC and OS in cohort 1 was initially analyzed. There was an association between MRE11 expression in CRC and OS (Figure S1A). Further results obtained by analyzing different CRC locations showed that high expression of MRE11 within the LSCRC was correlated with better OS, while expression of MRE11 within RSCC was not significant (Figure 2A). In a multivariate analysis, MRE11 expression remained a strong prognostic value, after adjusting for tumor stage, the grade of differentiation and TIICs (Table 1, HR = 0.37, 95% CI = 0.18–0.76, *P* = 0.007).

**Table 1:** Multivariate analyses of MRE11 expression for overall survival in patients with primary LSCRC from cohort 1.

**Parameters**	***P***	**HR**	**95% CI**	
			**Lower**	**Upper**
MRE11 expression (High vs. Low)	0.007	0.37	0.18	0.76
TIICs (High vs. Low)	0.002	0.37	0.19	0.69
TNM stages (I/II vs. III/IV)	<0.001	0.19	0.10	0.37
Grade (Well/Moderately vs. Poorly)	0.005	0.40	0.21	0.76

The prognostic significance of MRE11 in LSCRC was also found in the cohort 2 (Figure 2B), but no statistical significance was found in CRC and RSCC (Figure S1B and Figure 2B). Furthermore, high MRE11 expression in LSCRC was correlated with better OS in multivariate analysis after adjusting for stage (Table S3, HR = 0.46, 95% CI = 0.26–0.81, *P* = 0.007).

### The Prognostic Significance of the Combination of MRE11 With TIICs in RSCC and LSCRC

**Figure 3:**
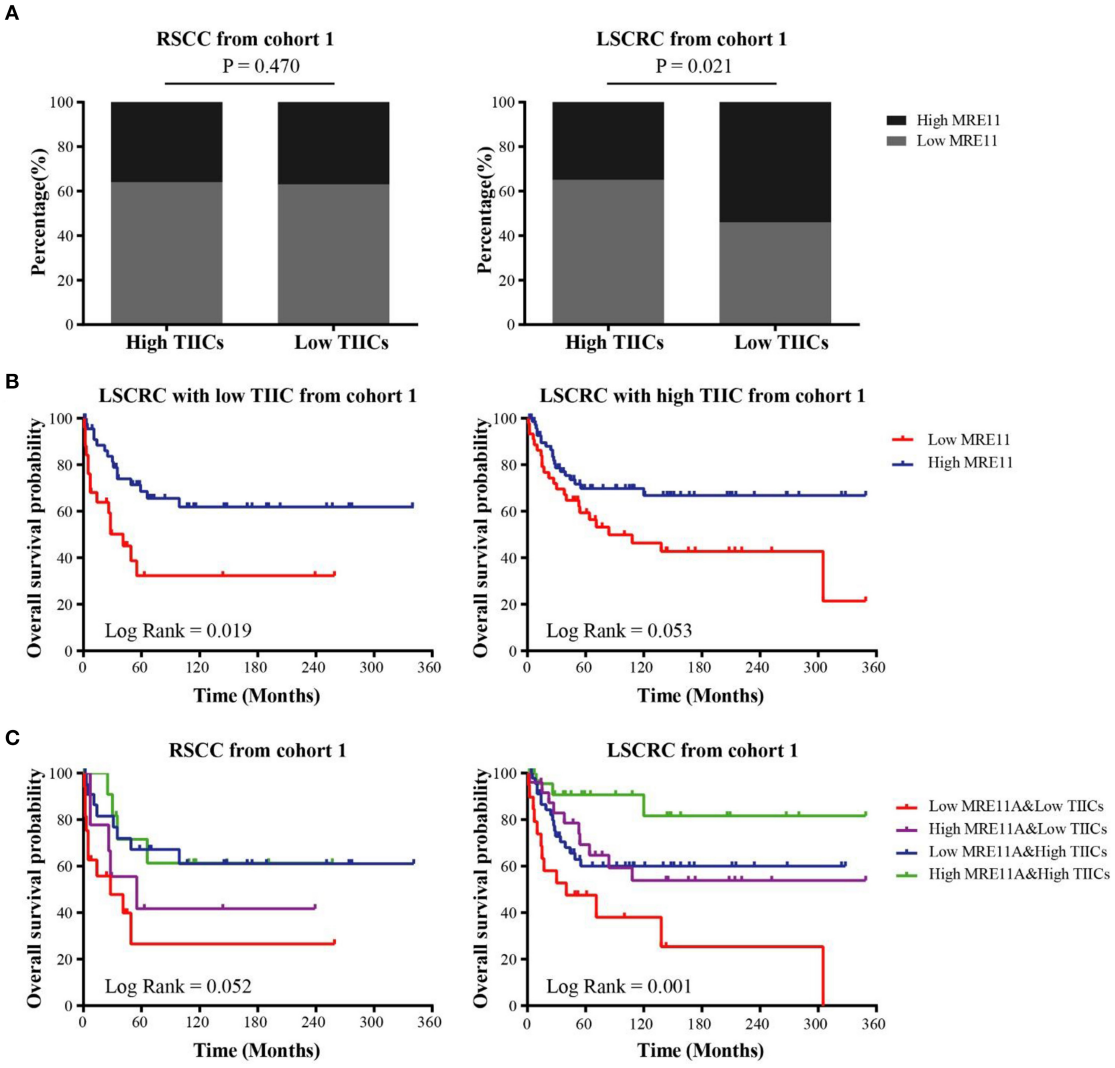
Association of MRE11 expression and TIICs with prognosis in RSCC and LSCRC. **(A)** MRE11 expression in RSCC (left panel) and LSCRC (right panel) tissue with high TIICs or low TIICs. **(B)** Kaplan-Meier OS curves of LSCRC patients with low TIICs (left panel) or high TIICs (right panel) stratified by MRE11 expression in the cohort 1. **(C)** Kaplan-Meier OS curves for the patients stratified by a combination of MRE11 and TIICs in RSCC (left panel) and LSCRC (right panel) in the cohort 1.

To understand the correlation between MRE11 expression and TIICs, as well as whether the subgroups of patients with a different prognosis can be identified, we investigated the MRE11 expression in primary tumor with high TIICs and low TIICs. As shown in Figure 3A, MRE11 expression was higher in LSCRC with low TIICs compared to high TIICs, while there were no significant differences in RSCC.

Furthermore, the OS of cohort 1 showed that there was also an association between MRE11 expression and TIICs in CRC. According to Figure 3B, high expression of MRE11 within low TIICs was correlated with better OS among patients with LSCRC. Similar trends were found in high TIICs (Figure 3B). Patients with high MRE11 expression and high TIICs had the best OS among the patients with LSCRC compared to patients with RSCC (Figure 3C). In multivariate analyses, high expression of MRE11 within high TIICs remained a strong prognostic value (Table 2, HR = 0.15, 95% CI = 0.04–0.52, *P* = 0.003), beyond the TNM stage (HR = 0.19, 95% CI = 0.1–0.37, *P* < 0.001) and the grade of differentiation (HR = 0.39, 95% CI = 0.20–0.76, *P* = 0.005).

**Table 2:** Multivariate analyses of the combination of MRE11 expression and TIICs for overall survival in LSCRC patients from cohort 1.

**Parameters**	***P***	**HR**	**95% CI**	
			**Lower**	**Upper**
High MRE11&High TIICs	0.003	0.15	0.04	0.52
High MRE11&Low TIICs	0.017	0.31	0.14	0.83
Low MRE11&High TIICs	0.005	0.35	0.17	0.72
Low MRE11&Low TIICs		1		
TNM stage (I/II vs. III/IV)	<0.001	0.19	0.1	0.37
Grade (Well/Moderately vs. Poorly)	0.005	0.39	0.20	0.76

### The Prognostic Significance of the Combination of MRE11 Expression With Microsatellite Status in RSCC and LSCRC

**Figure 4:**
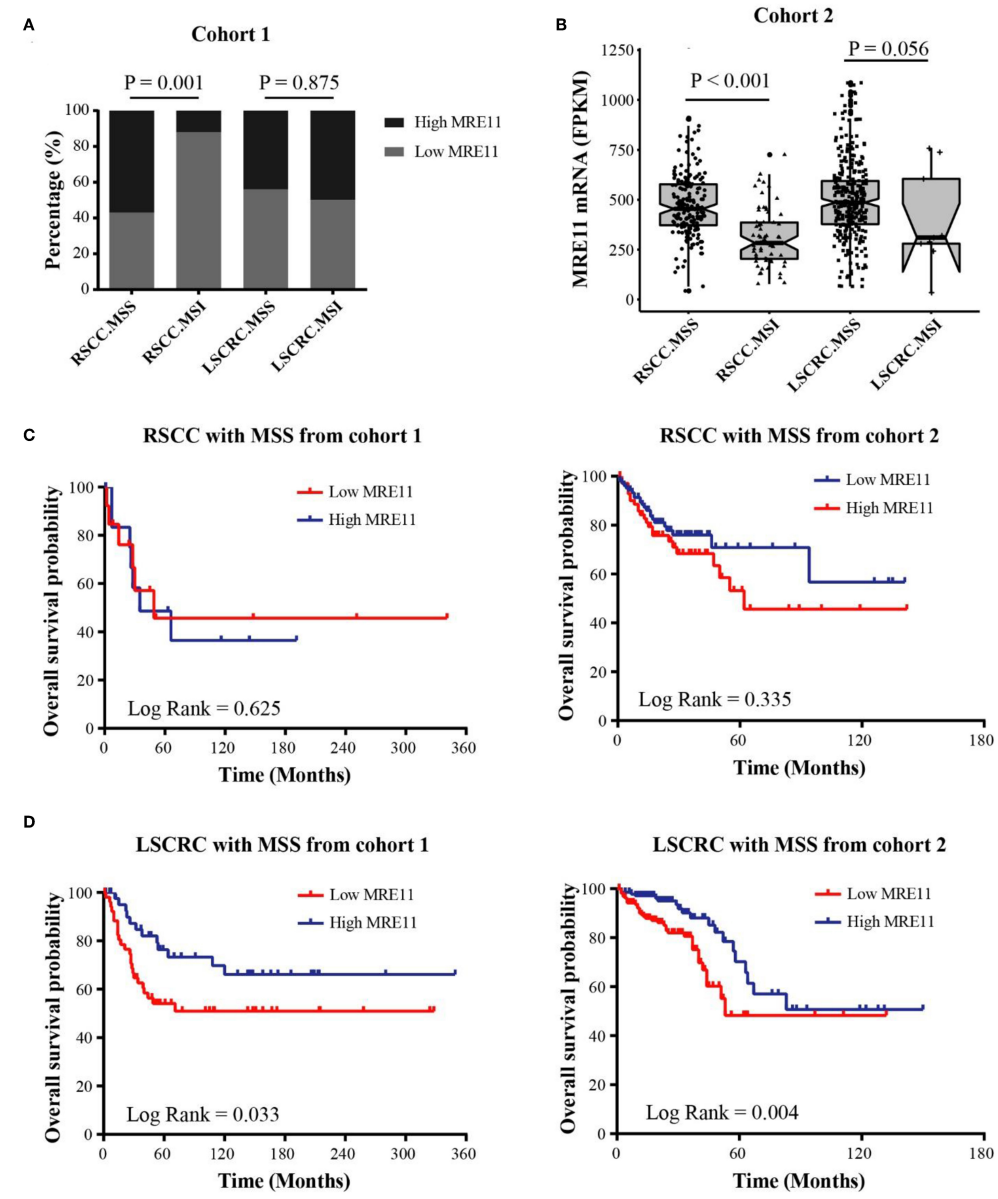
Association of MRE11 expression and MSI status with prognosis in RSCC and LSCRC. **(A)** MRE11 expression in RSCC and LSCRC tissue with MSS or MSI from cohort 1. **(B)** MRE11 mRNA expression in RSCC and LSCRC tissue with MSS or MSI from cohort 2. **(C)** Kaplan-Meier OS curves for RSCC patients with MSS stratified by MRE11 expression from cohort 1 (left panel) and cohort 2 (right panel). **(D)** Kaplan-Meier OS curves for LSCRC patients with MSS stratified by MRE11 expression from cohort 1 (left panel) and cohort 2 (right panel).

To explore the association between MRE11 expression and prognostic significance in RSCC or LSCRC with different microsatellite status, we separately investigated the MRE11 expression in MSS and MSI patients from cohort 1. According to the results, MRE11 expression was higher in RSCC with MSS compared to MSI (Figure 4A). No significant association between MRE11 expression and MSI status was observed in LSCRC (Figure 4A). These results were in line with the cohort 2 findings (Figure 4B).

We further analyzed the associations between MRE11 expression and OS in RSCC and LSCRC with different microsatellite status. The MRE11 expression in both protein and mRNA level had no survival significance among RSCC patient with either MSS or MSI (Figure 4C and Figures S2A,B). Nevertheless, patients with high MRE11 expression in both protein and mRNA level had a better OS compared to patients with low MRE11 expression in LSCRC with MSS (Figure 4D). Furthermore, by using multivariate analysis, similar outcomes for OS of LSCRC with MSS were observed in cohort 1 after adjusting for stage, the grade of differentiation and TIICs (Table 3, HR = 0.23, 95% CI = 0.09–0.55, *P* = 0.001), and in cohort 2 after adjusting for stage, respectively (Table S4, HR = 0.41, 95% CI = 0.23–0.74, *P* = 0.003).

**Table 3:** Multivariate analyses of MRE11 expression for overall survival in LSCRC with MSS from cohort 1.

**Parameters**	***P***	**HR**	**95% CI**	
			**Lower**	**Upper**
MRE11 expression (High vs. Low)	0.001	0.23	0.09	0.55
TIICs (High vs. Low)	0.001	0.25	0.11	0.56
TNM stages (I/II vs. III/IV)	<0.001	0.13	0.06	0.30
Grade (Well/Moderately vs. Poorly)	0.172	0.59	0.27	1.26

### WGCNA Identified Different Networks Associated With MRE11 Expression in RSCC and LSCRC

**Figure 5:**
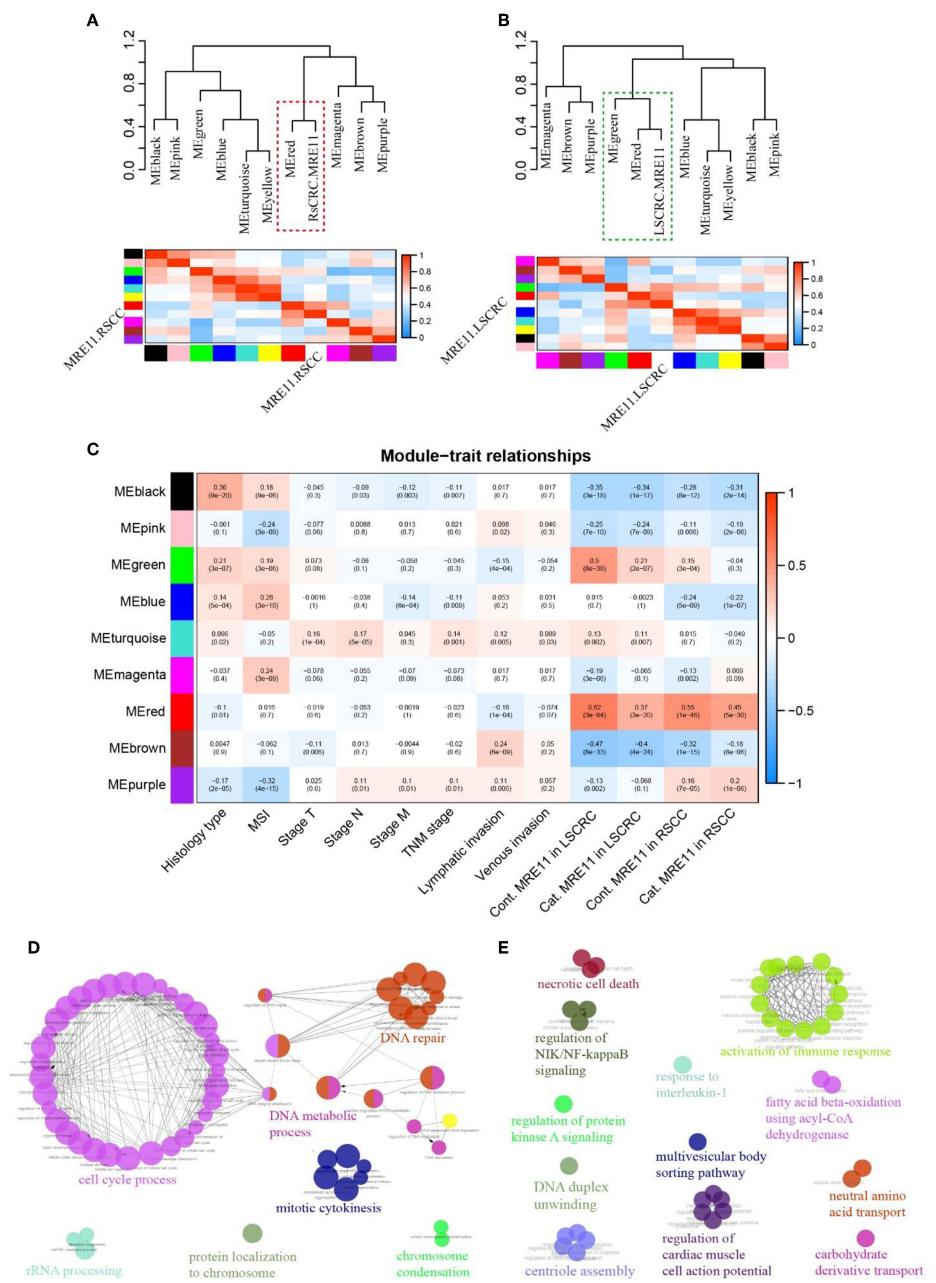
MRE11-related gene enrichment and annotation in RSCC and LSCRC based on WGCNA. **(A)** Eigengene network, including the clustering tree and heatmap, represents the relationships among the modules and the MRE11 expression in RSCC. The red box indicates modules that strongly correlated with MRE11 expression in RSCC. **(B)** Eigengene network, including the clustering tree and heatmap, represents the relationships among the modules and the MRE11 expression in LSCRC. The green box indicates modules that strongly correlation with MRE11 expression in LSCRC. **(C)** Pearson correlation coefficient between the module eigengene of modules and the clinical feature. Numbers in rectangular represent the correlation coefficients and numbers in brackets indicate the corresponding *P*-values. Cont., continuous variable; Cat., categorical variable. **(D)** Enriched functions and pathways of the MRE11-related red module genes common in both of RSCC and LSCRC. The pathways are functionally grouped and interconnected based on the kappa score. The size of the nodes shows the term significance after Bonferroni correction (*P* < 0.01). **(E)** Enriched functions and pathways of the MRE11-related green module genes in LSCRC. The pathways are functionally grouped and interconnected based on the kappa score. The size of the nodes shows the term significance after Benjamini-Hochberg correction (*P* < 0.05).

To investigate whether the function of MRE11 involved in LSCRC is different from RSCC, WGCNA was employed. Co-expression network was constructed using DEGs based on cohort 2 data, and clinical traits were included in the present analysis. We examined the association between several gene expression modules, including MRE11 expression in RSCC and LSCRC, as main parameters. Interestingly, MRE11 expression in RSCC and LSCRC clustered with a different set of gene modules (Figures 5A,B). In parallel, we examined the association between each of the modules and some clinicopathological traits, including MRE11 expression, histological type, microsatellite status, lymphatic invasion, venous invasion, and TNM stage. Most of the clinicopathological traits, which have an impact on gene expression were independent of each other (Figure 5C). Regarding modules of MRE11 expression in RSCC and LSCRC, it was noticed that some of them were correlated with one phenotype but not the others, while some modules correlated both with MRE11 expression in RSCC and LSCRC (Figure 5C). We further examined the specific modules (red and green) that showed the strongest correlation with MRE11 expression (Figure 5C and Figures S3B,C).

In order to understand the role of these modules in depth, we performed ClueGo analysis on the genes. The red module strongly clustered with both MRE11 in RSCC and LSCRC. Genes enriched in this module were mainly involved in the cell cycle process, DNA repair, DNA metabolic process and mitotic cytokinesis (Figure 5D and Table S5). However, the green module strongly clustered with MRE11 as a continuous variable in LSCRC (*r* = 0.5, *P* = 8e-38) and only moderately in RSCC (*r* = 0.15, *P* = 3e-4) (Figure 5C and Figure S3C). Meanwhile, the green module strongly correlated with MRE11 as a categorical variable in LSCRC (*r* = 0.21, *P* = 2e-7) and no correlation was found in RSCC (*r* = −0.04, *P* = 0.3) (Figure 5C and Figure S3C). The genes involved in this module were mainly associated with activation of the immune response, response to interleukin-1, necrotic cell death, regulation of NIK/NF-kappa B signaling and regulation of protein kinase A signaling (Figure 5E and Table S6).

## Discussion

MRE11 expression is becoming a prognostic marker in several types of tumors, including CRC, while its role is still a subject of debate (13, 14, 23). Whether MRE11 expression in different tumor location of CRC has a significant prognostic impact is yet to be manifested. In the present study, we analyzed the MRE11 expression and prognosis in different tumor location. The results have shown that MRE11 expression is significantly increased in CRC tissue compared to normal tissue (19). Most importantly, its expression in LSCRC is greater rather than in RSCC. The differential expression of MRE11 indicates a diverse role within RSCC and LSCRC, similar to the higher expression of chromosomal instability (CIN) in LSCRC compared to RSCC (24). Our previous findings have shown that MRE11 was a potential prognostic factor in CRC (19). In this study, by analyzing the prognostic value in RSCC and LSCRC, high expression of MRE11 was found to be a favorable factor for prognosis in LSCRC rather than RSCC, which underlines that the survival benefit of the high expression of MRE11 is not derived from any CRC location, but the LSCRC. This result is a novel finding that enhances the molecular characteristics of LSCRC, which is same as CIN, p53, and NRAS (24).

Apart from distinct clinical and genomic features between RSCC and LSCRC, MSI and MSS tumors are also regarded as two different heterogeneous entities. More specifically, patients with MSI tumors have longer overall and cancer-specific survival compared to patients with MSS tumors (25, 26). However, MSI is more commonly located on RSCC (15). In parallel, we analyzed the prognostic significance of MRE11 in RSCC and LSCRC with MSS or MSI. Patients with high MRE11 expression for both protein and mRNA level had a statistically better significance of OS compared to low MRE11 expression in LSCRC with MSS. No significant difference of OS was present in RSCC with MSS. The present results indicate that this difference is mainly derived from LSCRC with MSS rather than RSCC. However, we cannot significantly evaluate the correlation between clinical features and MSI tumor in LSCRC, as this study included only 6 MSI and 9 MSI patients with LSCRC among cohort 1 and cohort 2 data, which is a low number of patients in order to be further explored.

By identifying the MSI phenotype and the corresponding prognostic advantage, the host immune response, and the enhanced lymphocytic reaction, has become the main focus of the current cancer study (22). The presence of TIICs is more common in MSI rather than MSS tumors (27). Our data demonstrated that the MRE11 expression is different in LSCRC with high or low TIICs, while no significance was present in RSCC. When the two markers were combined, the patients with very good prognosis (high MRE11 expression and high TIICs) or very poor prognosis (low MRE11 expression and low TIICs) were well-distinguished in LSCRC. However, the link between MRE11 and tumor-infiltrating lymphocytes (TILs) had no significant prognostic value above TILs in anal cancer, which is the only study describing the combination of MRE11 and TILs up to date (28). This outcome suggests that the correlation between MRE11 and TIICs differs among cancers of different tissue origin, and the differential clinical significance of combination between MRE11 and TIICs in the cancers from same organ which have different embryo origin (e.g., RSCC and LSCRC), although we could not detect the specific types of TIICs in the present study. Overall, the above results strongly suggest that patient survival was MRE11 and TIICs dependent which can become a novel prognostic marker in LSCRC.

Apart from DNA mismatch repair, deficiencies cause frameshift mutations and produce immunogenic neoantigens in MSI tumor (29), little is known regarding the link between MRE11 expression and TIICs in CRC. Thus, we tried to reveal the potential mechanism by using the WGCNA which is a powerful tool to enrich signal network (21). Our data has shown that the MRE11-related gene clusters are differentially enriched in RSCC and LSCRC. MRE11 expression is strongly correlated with activation of immune response, response to interleukin-1, necrotic cell death, regulation of NIK/NF-kappa B signaling and regulation of protein kinase A signaling in LSCRC, indicating that combination of MRE11 with TIICs is a strong predictive factor of OS. This can be mainly due to MRE11 involvement in the activation of immune-related signaling. Similarly, cytotoxic T-cells, natural killer, dendritic cells, nuclear factor-κB signatures are overexpressed in CIN-low triple-negative breast cancer, which is characterized by an intense immune infiltration and overall good prognosis (30). Although we did not further verify the MRE11-activated immune pathway by an experimental approach in the current study, we provided an evidence that there was an obvious difference in activation of immunosignals between RSCC and LSCRC. The DDR not only directly linked to innate immunity, as cells are adept at sensing damaged and foreign DNA, but also the DDR can mediate response to immunotherapy by interaction with immune system (17). Even, DNA repair factors affect antitumor immunity beyond mutational load. Therefore, the MRE11, as a crucial protein in DNA damage/repair pathway, perhaps could be a potential predictive biomarker for response to immunotherapy. However, the mechanism, why the MRE11 plays different roles in the activation of immunosignals between RSCC and LSCRC, needs to be clarified in the future work.

In conclusion, our findings have revealed that high MRE11 expression combined with or without high TIICs, is related to a favorable prognosis in LSCC beyond TNM stage. Moreover, our results suggest that the cell signaling, especially immune response activated by MRE11 may differ in RSCC and LSCRC. These heterogeneities provide a novel rationale for CRC patient stratification in the future, which can potentially lead to a more accurate and prognostic patient evaluation.

## Data Availability Statement

The current study includes data from the two cohorts, the cohort 1 which is our own immunohistochemistrial data and the cohort 2 which is TCGA data. All original data in the cohort 1 for this study is included in the article/Supplementary Material. The cohort 2 is openly obtained from UCSC Xena datasets (https://xenabrowser.net/datapages/).

## Ethics Statement

The cohort 1 of this study was approved by the Research Ethics Committee of Linkoping University (Dnr 2012-107-31). All patients gave written informed consent prior to specimen collection according to Linkoping University in accordance with the Declaration of Helsinki. The cohort 2 is TCGA data which is in accordance with these ethical and legal guidelines by TCGA Ethics and Policies published by the National Cancer Institute.

## Author Contributions

CWF, ZGZ, and XFS: conceptualization. CWF, MK, YBL, JFG, and HZ: methodology. CWF, YBL, JFG, HZ, and GA: formal analysis. CWF and SYWC: data curation. CWF and MK: writing original draft preparation. YL, ZGZ, and XFS: writing review and editing. HZ, YL, ZGZ, and XFS: supervision.

### Conflict of Interest

The authors declare that the research was conducted in the absence of any commercial or financial relationships that could be construed as a potential conflict of interest.
