# Supplementary material for: Prognostic Heterogeneity of MRE11 Based on the Location of Primary Colorectal Cancer Is Caused by Activation of Different Immune Signals
Source: Front Oncol. 2020 Jan 17;9:1465. doi: 10.3389/fonc.2019.01465 (PMC6979908; doi:10.3389/fonc.2019.01465)
Supplement: Supplementary file 1 [file Data_Sheet_1.docx]

**Supplementary Materials**

1. **Supplementary Figures**

**Figure S1**. Association of MRE11 expression with clinical outcome in CRC.

**Figure S2**. Association of MRE11 expression with clinical outcome in CRC with MSI.

**Figure S3.** Identification of modules associated with the MRE11 expression in RSCC and LSCRC.

1. **Supplementary Tables**

**Table S1.** MRE11 expression and clinicopathological parameters in CRC and its locations from cohort 1

**Table S2**. MRE11 expression and clinicopathological parameters in CRC and its locations from cohort 2

**Table S3**. Multivariate analyses of MRE11 expression for overall survival in patients with primary LSCRC from cohort 2

**Table S4.** Multivariate analyses of MRE11 expression for overall survival in LSCRC with MSS from cohort 2

**Table S5.** ClueGO-CluePedia functional analysis of red module from cohort 2.

**Table S6.** ClueGO-CluePedia functional analysis of green module in LSCRC from cohort 2.

1. **Supplementary Methods**
2. **Supplementary Figures**

**
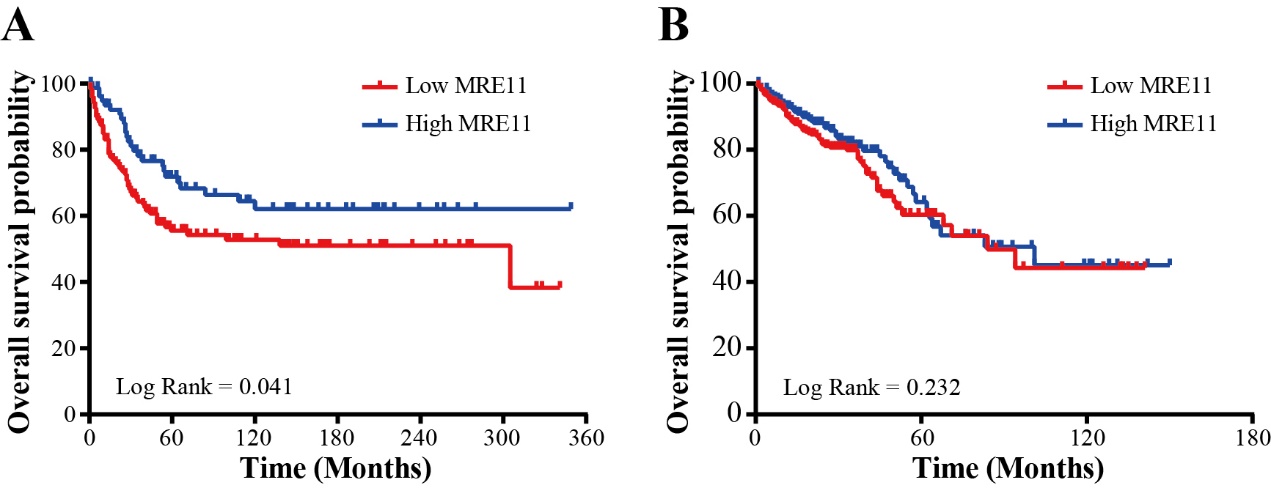
**

**Figure S1**. **Association of MRE11 expression with clinical outcome in CRC.** (A) Kaplan-Meier OS curves of 207 CRC patients from cohort 1. (B) Kaplan-Meier OS curves of 596 CRC patients from cohort 2.


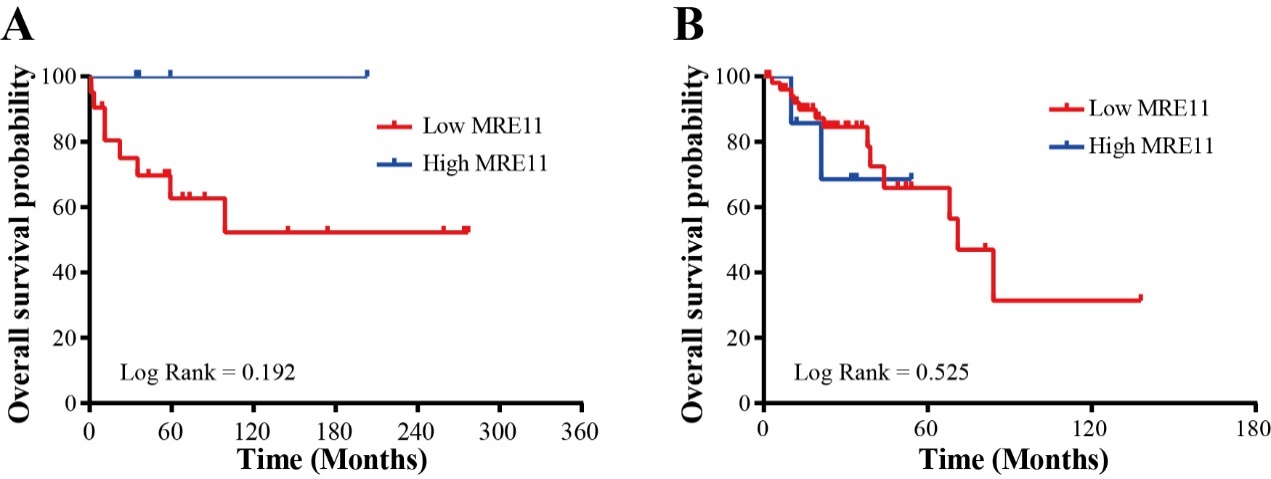


**Figure S2**. **Association of MRE11 expression with clinical outcome in CRC with MSI.** (A) Kaplan-Meier overall survival curves of 22 RSCC patients with MSI from cohort 1. (B) Kaplan-Meier OS curves of 58 RSCC patients with MSI from cohort 2.


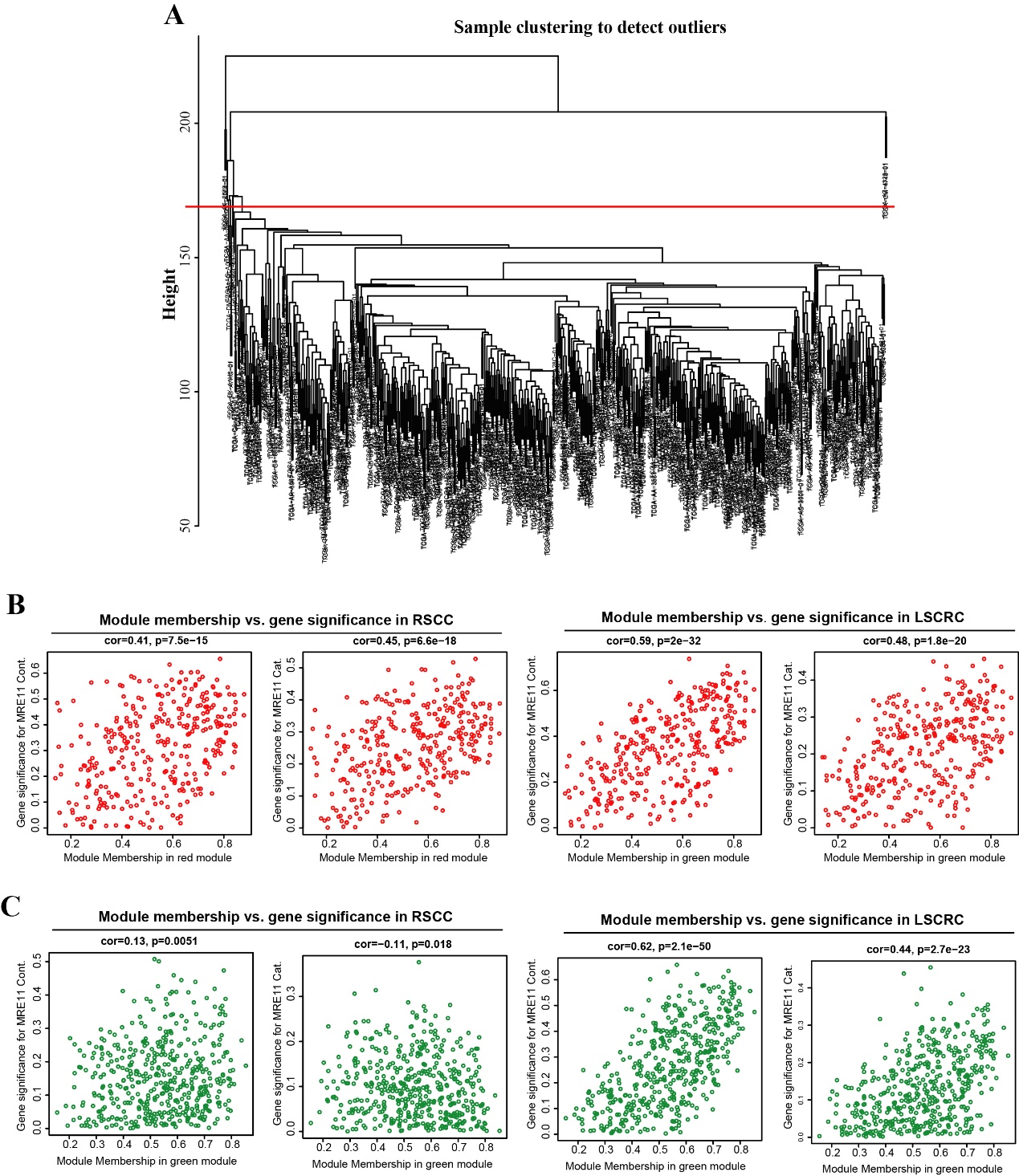


**Figure S3. Identification of modules associated with the MRE11 expression in RSCC and LSCRC.** (A) Clustering dendrogram of 596 primary CRC samples from cohort 2. Red line indicated the cutoff point (165) to remove the outlier samples. (B) Scatter plot for correlation between gene module membership and gene significance of MRE11 expression as a continuous (cont.) or categorical (cat.) variable in the red module (MRE11-module) from RSCC. (C) Scatter plot for correlation between gene module membership and gene significance of MRE11 expression as a continuous (cont.) or categorical (cat.) variable in the green module from LSCRC.

1. **Supplementary Tables**

**Table S1.** MRE11 expression and clinicopathological parameters in CRC and its locations from cohort 1

| Parameters | | MRE11 expression | | | | | |
| --- | --- | --- | --- | --- | --- | --- | --- |
|  |  | CRC (207) | | RSCC (74) | | LSCRC (132) | |
|  |  | low (125) | high (82) | low (49) | high (25) | low (75) | high (57) |
| Gender | Male | 71 (56.8) | 43 (52.4) | 29 (59.2) | 12 (48.0) | 42 (56.0) | 31 (54.4) |
|  | Female | 54 (43.2) | 39 (47.6) | 20 (40.8) | 13 (52.0) | 33 (44.0) | 26 (45.6) |
| Age (years) | <70 | 42 (33.6) | 35 (42.7) | 17 (34.7) | 8 (32.0) | 24 (32.0) | 27 (47.4) |
|  | ≥70 | 83 (66.4) | 47 (57.3) | 32 (65.3) | 17 (68.0) | 51 (68.0) | 30 (52.6) |
| Location | RSCRC | 49 (39.2) | 25 (30.5) | NA | NA | NA | NA |
|  | LSCRC | 75 (60.0) | 57 (69.5) | NA | NA | NA | NA |
|  | Missing data | 1 (0.8) | 0 (0.0) | NA | NA | NA | NA |
| TNM stage | I | 12 (9.6) | 13 (15.9) | 1 (2.0) | 2 (8.0) | 11 (14.7) | 11 (19.3) |
|  | II | 40 (32.0) | 34 (41.5) | 17 (34.7) | 9 (36.0) | 23 (30.7) | 25 (43.9) |
|  | III | 45 (36.0) | 18 (22.0) | 18 (36.7) | 6 (24.0) | 27 (36) | 12 (21.1) |
|  | IV | 26 (20.8) | 15 (18.3) | 11 (22.4) | 7 (28.0) | 14 (18.7) | 8 (14.0) |
|  | Missing data | 2 (1.6) | 2 (2.4) | 2 (4.1) | 1 (4.0) | 0 (0.0) | 1 (1.8) |
| Growth Pattern | Expansion | 65 (52) | 44 (53.7) | 22 (44.9) | 12 (48.0) | 43 (57.3) | 32 (56.1) |
|  | Infiltration | 52 (41.6) | 34 (41.5) | 21 (42.9) | 10 (40.0) | 30 (40) | 24 (42.1) |
| Histological types | Adenocarcinoma | 105 (84.0) | 69 (84.1) | 39 (79.6) | 20 (80.0) | 65 (86.7) | 49 (86.0) |
|  | Mucinous adenocarcinoma | 20 (16.0) | 13 (15.9) | 10 (20.4) | 5 (20.0) | 10 (13.3) | 8 (14.0) |
| Grade | Well | 9 (7.2) | 3 (3.7) | 2 (4.1) | 0 (0.0) | 7 (9.3) | 3 (5.3) |
|  | Moderately | 77 (61.6) | 54 (65.9) | 27 (55.1) | 16 (64.0) | 50 (66.7) | 38 (66.7) |
|  | Poorly | 39 (31.2) | 25 (30.5) | 20 (40.8) | 9 (36.0) | 18 (24) | 16 (28.1) |
|  | Missing data | 8 (6.4) | 4 (4.9) | 6 (12.2) | 3 (12.0) | 2 (2.7) | 1 (1.8) |
| TIICs | Low | 38 (30.4) | 35 (42.7) | 17 (34.7) | 10 (40.0) | 21 (28.0) | 25 (43.9) |
|  | High | 70 (56.0) | 38 (46.3) | 23 (46.9) | 13 (52.0) | 46 (61.3) | 25 (43.9) |
|  | Missing data | 17 (13.6) | 9 (11.0) | 9 (18.4) | 2 (8.0) | 8 (10.7) | 7 (12.3) |
| MSI status | MSS | 68 (54.4) | 60 (73.2) | 12 (24.5) | 16 (64.0) | 55 (73.3) | 44 (77.2) |
|  | MSI | 25 (20.0) | 6 (7.3) | 22 (44.9) | 3 (12.0) | 3 (4.0) | 3 (5.3) |
|  | Missing data | 32 (25.6) | 16 (19.5) | 15 (30.6) | 6 (24.0) | 17 (22.7) | 10 (17.5) |
| Recurrence | No | 39 (31.2) | 34 (41.5) | 11 (22.4) | 10 (40.0) | 28 (37.3) | 24 (42.1) |
|  | YES | 9 (7.2) | 11 (13.4) | 5 (10.2) | 3 (12.0) | 4 (5.3) | 8 (14.0) |
|  | Missing data | 77 (61.6) | 37 (45.1) | 33 (67.3) | 12 (48.0) | 43 (57.3) | 25 (43.9) |

**Table S2**. MRE11 expression and clinicopathological parameters in CRC and its locations from cohort 2

| Parameters | | MRE11 expression | | | | | |
| --- | --- | --- | --- | --- | --- | --- | --- |
|  |  | CRC (596) | | RSCC (251) | | LSCRC (328) | |
|  |  | low (358) | high (238) | low (169) | high (82) | low (179) | high (149) |
| Gender | Male | 174 (48.6) | 101 (42.4) | 78 (46.2) | 38 (46.3) | 90 (50.3) | 61 (40.9) |
|  | Female | 184 (51.4) | 137 (57.6) | 91 (53.8) | 44 (53.7) | 89 (49.7) | 88 (59.1) |
| Age (years) | <70 | 197 (55.0) | 153 (64.3) | 84 (49.7) | 41 (50) | 108 (60.3) | 106 (71.1) |
|  | ≥70 | 161 (45.0) | 85 (35.7) | 85 (50.3) | 41 (50) | 71 (39.7) | 43 (28.9) |
| Location | RSCRC | 169 (47.2) | 82 (34.5) | NA | NA | NA | NA |
|  | LSCRC | 179 (50.0) | 149 (62.6) | NA | NA | NA | NA |
|  | Missing data | 10 (2.8) | 7 (2.9) | NA | NA | NA | NA |
| TNM stages | I | 67 (18.7) | 38 (16.1) | 28 (16.6) | 17 (20.7) | 37 (20.7) | 21 (14.1) |
|  | II | 233 (65.1) | 161 (67.6) | 123 (72.8) | 53 (64.6) | 103 (57.5) | 104 (69.8) |
|  | III | 51 (14.2) | 34 (14.3) | 15 (8.9) | 10 (12.2) | 35 (19.6) | 21 (14.1) |
|  | IV | 7 (2.0) | 5 (2.1) | 3 (1.8) | 2 (2.4) | 4 (2.2) | 3 (2.0) |
| Histological types | Adenocarcinoma | 292 (81.6) | 220 (92.4) | 133 (78.7) | 70 (85.4) | 152 (84.9) | 144 (96.6) |
|  | Mucinous adenocarcinoma | 56 (15.6) | 17 (7.2) | 35 (20.7) | 12 (14.6) | 20 (11.2) | 5 (3.4) |
|  | Missing data | 10 (2.8) | 1 (0.4) | 1 (0.6) | 0 (0.0) | 7 (3.9) | 0 (0) |
| Lymphatic invasion | No | 194 (54.2) | 130 (54.6) | 96 (56.8) | 50 (61.0) | 92 (51.4) | 76 (51.0) |
|  | Yes | 137 (38.3) | 75 (31.5) | 57 (33.7) | 22 (26.8) | 76 (42.5) | 50 (33.6) |
|  | Missing data | 27 (7.5) | 33 (13.9) | 16 (9.5) | 10 (12.2) | 11 (6.1) | 23 (15.4) |
| Venous invasion | No | 234 (65.4) | 157 (66.0) | 113 (66.9) | 52 (63.4) | 114 (63.7) | 100 (67.1) |
|  | Yes | 77 (21.5) | 48 (20.2) | 27 (16.0) | 22 (26.8) | 47 (26.3) | 25 (16.8) |
|  | Missing data | 47 (13.1) | 33 (13.8) | 29 (17.1) | 8 (9.8) | 18 (10.0) | 24 (16.1) |
| Perineural invasion | No | 83 (23.2) | 84 (35.3) | 40 (26.0) | 31 (37.8) | 34 (19.0) | 53 (35.6) |
|  | Yes | 29 (8.1) | 29 (12.2) | 19 (11.2) | 11 (13.4) | 8 (4.5) | 18 (12.1) |
|  | Missing data | 246 (68.7) | 125 (52.5) | 106 (62.8) | 40 (48.8) | 137 (76.5) | 78 (52.3) |
| MSI status | MSS | 283 (79.0) | 222 (93.3) | 109 (64.5) | 71 (86.6) | 169 (94.4) | 144 (96.6) |
|  | MSI | 68 (19.0) | 11 (4.6) | 58 (34.3) | 8 (9.8) | 6 (3.4) | 3 (2.0) |
|  | Missing data | 7 (2.0) | 5 (2.1) | 2 (1.2) | 3 (3.7) | 3 (1.7) | 2 (1.3) |
| Recurrence | No | 233 (65.1) | 160 (67.2) | 115 (68.0) | 56 (68.3) | 110 (61.5) | 101 (67.8) |
|  | Yes | 53 (14.8) | 53 (22.3) | 22 (13.0) | 19 (23.2) | 29 (16.2) | 30 (20.1) |
|  | Missing data | 72 (20.1) | 25 (10.5) | 32 (19.0) | 7 (8.5) | 40 (22.3) | 18 (12.1) |

**Table S3**. Multivariate analyses of MRE11 expression for overall survival in patients with primary LSCRC from cohort 2

| Parameters | *P* | HR | 95% CI | |
| --- | --- | --- | --- | --- |
|  |  |  | Lower | Upper |
| MRE11 expression (High *vs* Low) | 0.007 | 0.46 | 0.26 | 0.81 |
| TNM stage (I/II *vs* III/IV) | < 0.001 | 0.27 | 0.15 | 0.50 |

**Table S4.** Multivariate analyses of MRE11 expression for overall survival in LSCRC with MSS from cohort 2

| Parameters | *P* | HR | 95% CI | |
| --- | --- | --- | --- | --- |
|  |  |  | Lower | Upper |
| MRE11 expression (High *vs* Low) | 0.003 | 0.41 | 0.23 | 0.74 |
| TNM stage (I/II *vs* III/IV) | < 0.001 | 0.25 | 0.13 | 0.47 |

**Table S5.** ClueGO-CluePedia functional analysis of red module from cohort 2.

| GO Functional Groups | Group *P* Value | GO Term | Term *P* Value | Associated Genes Found |
| --- | --- | --- | --- | --- |
| cell cycle process | 7.52E-18 | cell cycle process | 7.26E-17 | [ANLN, ASPM, ATAD5, AUNIP, AURKA, BLM, BRCA1, BRCA2, BRIP1, BUB1, CCNA2, CDC25C, CDC6, CDCA5, CDK1, CDKN3, CENPA, CENPE, CENPF, CEP55, CHEK1, CKAP2, CLSPN, DEPDC1B, DONSON, DSCC1, DTL, E2F7, ECT2, EZH2, FANCD2, FZD3, HSP90AB1, INTS13, KIF14, KIF18A, KIF23, KIF2C, KIF4A, KNTC1, MAD2L1, MKI67, NCAPG, NCAPH, NEK2, NUSAP1, POLR1B, PRKDC, PTK2B, RACGAP1, SGO1, SPC25, STIL, TMEM67, TOM1L2, TPX2, TRIP13, UBE2C, XRCC2] |
| cell cycle process | 7.52E-18 | mitotic cell cycle process | 1.01E-16 | [ANLN, BLM, BRCA2, BRIP1, CCNA2, CDC25C, CDC6, CDCA5, CDK1, CENPA, CENPE, CENPF, CEP55, CHEK1, CKAP2, CLSPN, DONSON, DSCC1, DTL, E2F7, ECT2, FANCD2, INTS13, KIF14, KIF18A, KIF23, KIF2C, KIF4A, KNTC1, MAD2L1, MKI67, NCAPG, NCAPH, NEK2, NUSAP1, POLR1B, PRKDC, PTK2B, RACGAP1, SGO1, SPC25, STIL, TOM1L2, TPX2, TRIP13, UBE2C] |
| cell cycle process | 7.52E-18 | mitotic cell cycle | 2.99E-16 | [ANLN, BLM, BRCA2, BRIP1, CCNA2, CDC25C, CDC6, CDCA5, CDK1, CENPA, CENPE, CENPF, CEP55, CHEK1, CKAP2, CLSPN, DONSON, DSCC1, DTL, E2F7, ECT2, FANCD2, INTS13, KIF14, KIF18A, KIF23, KIF2C, KIF4A, KNTC1, MAD2L1, MKI67, NCAPG, NCAPH, NEK2, NUSAP1, POLR1B, PRKDC, PTK2B, RACGAP1, SGO1, SKA1, SKA3, SPC25, STIL, TOM1L2, TPX2, TRIP13, UBE2C, XRCC2] |
| cell cycle process | 7.52E-18 | mitotic nuclear division | 5.03E-08 | [CDC6, CDCA5, CENPE, CHEK1, DSCC1, KIF14, KIF18A, KIF23, KIF2C, KIF4A, MAD2L1, MKI67, NCAPG, NCAPH, NEK2, NUSAP1, RACGAP1, SGO1, TOM1L2, TPX2, TRIP13, UBE2C] |
| cell cycle process | 7.52E-18 | nuclear chromosome segregation | 5.75E-08 | [BUB1, CDC6, CDCA5, CENPE, CENPF, DSCC1, ECT2, KIF14, KIF18A, KIF23, KIF2C, KIF4A, MAD2L1, NCAPG, NCAPH, NEK2, NUSAP1, RACGAP1, SGO1, TRIP13] |
| cell cycle process | 7.52E-18 | nuclear division | 1.29E-07 | [CDC6, CDCA5, CENPE, CHEK1, DSCC1, KIF14, KIF18A, KIF23, KIF2C, KIF4A, MAD2L1, MKI67, NCAPG, NCAPH, NEK2, NUSAP1, RACGAP1, SGO1, TOM1L2, TPX2, TRIP13, UBE2C] |
| cell cycle process | 7.52E-18 | cell cycle phase transition | 2.01E-07 | [ATAD5, BLM, BRCA1, BRIP1, CCNA2, CDC25C, CDC6, CDCA5, CDK1, CENPE, CENPF, CHEK1, CLSPN, DEPDC1B, DONSON, DTL, E2F7, EZH2, KIF14, MAD2L1, POLR1B, PRKDC, PTK2B, TRIP13, UBE2C] |
| cell cycle process | 7.52E-18 | organelle fission | 2.81E-07 | [CDC6, CDCA5, CENPE, CHEK1, DSCC1, KIF14, KIF18A, KIF23, KIF2C, KIF4A, MAD2L1, MKI67, NCAPG, NCAPH, NEK2, NUSAP1, PINK1, RACGAP1, SGO1, TOM1L2, TPX2, TRIP13, UBE2C] |
| cell cycle process | 7.52E-18 | regulation of cell cycle phase transition | 2.87E-07 | [ATAD5, BLM, BRCA1, CDC6, CDCA5, CDK1, CENPE, CENPF, CHEK1, CLSPN, DEPDC1B, DONSON, DTL, EZH2, KIF14, MAD2L1, POLR1B, PRKDC, PTK2B, TRIP13, UBE2C] |
| cell cycle process | 7.52E-18 | regulation of cell cycle process | 3.21E-07 | [ATAD5, BLM, BRCA1, BUB1, CDC6, CDCA5, CDK1, CENPE, CENPF, CHEK1, CLSPN, DEPDC1B, DONSON, DTL, ECT2, EZH2, FZD3, HSP90AB1, KIF14, KIF23, MAD2L1, MKI67, NEK2, NUSAP1, POLR1B, PRKDC, PTK2B, RACGAP1, STIL, TMEM67, TOM1L2, TRIP13, UBE2C] |
| cell cycle process | 7.52E-18 | chromosome organization | 7.91E-07 | [BLM, BRCA1, BRCA2, BUB1, CCNA2, CCT6A, CDC6, CDCA5, CDKN3, CENPA, CENPE, CHAF1B, DEPDC1B, DKC1, DSCC1, EZH2, FANCD2, GNL3, HJURP, HSP90AB1, JADE3, KIF14, KIF18A, KIF23, KIF2C, KIF4A, MAD2L1, MPP7, NAT10, NCAPG, NCAPH, NEK2, NUSAP1, POLR1B, PRKDC, PRMT3, RACGAP1, SGO1, SUV39H2, TOP2A, TRIP13] |
| cell cycle process | 7.52E-18 | mitotic sister chromatid segregation | 1.35E-06 | [CDC6, CDCA5, CENPE, DSCC1, KIF14, KIF18A, KIF23, KIF2C, KIF4A, MAD2L1, NCAPG, NCAPH, NUSAP1, RACGAP1, SGO1, TRIP13] |
| cell cycle process | 7.52E-18 | sister chromatid segregation | 2.00E-06 | [BUB1, CDC6, CDCA5, CENPE, DSCC1, KIF14, KIF18A, KIF23, KIF2C, KIF4A, MAD2L1, NCAPG, NCAPH, NUSAP1, RACGAP1, SGO1, TRIP13] |
| cell cycle process | 7.52E-18 | regulation of cell cycle | 4.07E-06 | [ATAD5, BLM, BRCA1, BUB1, CDC6, CDCA5, CDK1, CDKN3, CENPE, CENPF, CHEK1, CLSPN, DEPDC1B, DNA2, DONSON, DTL, ECT2, EZH2, FZD3, GTPBP4, HSP90AB1, INTS13, KIF14, KIF23, KNTC1, MAD2L1, MKI67, NEK2, NUSAP1, POLR1B, PRKDC, PRR11, PTK2B, RACGAP1, STIL, TMEM67, TOM1L2, TRIP13, UBE2C] |
| cell cycle process | 7.52E-18 | cell cycle G2/M phase transition | 4.26E-06 | [ATAD5, BLM, BRCA1, BRIP1, CCNA2, CDC25C, CDK1, CENPF, CHEK1, CLSPN, DEPDC1B, DONSON, DTL, KIF14] |
| cell cycle process | 7.52E-18 | mitotic cell cycle phase transition | 2.23E-05 | [BLM, BRIP1, CCNA2, CDC25C, CDC6, CDCA5, CDK1, CENPE, CENPF, CLSPN, DONSON, DTL, E2F7, KIF14, MAD2L1, POLR1B, PRKDC, PTK2B, TRIP13, UBE2C] |
| cell cycle process | 7.52E-18 | regulation of cell cycle G2/M phase transition | 6.15E-05 | [ATAD5, BLM, BRCA1, CDK1, CENPF, CHEK1, CLSPN, DEPDC1B, DONSON, DTL, KIF14] |
| cell cycle process | 7.52E-18 | regulation of mitotic cell cycle phase transition | 1.03E-04 | [BLM, CDC6, CDCA5, CDK1, CENPE, CENPF, CLSPN, DONSON, DTL, KIF14, MAD2L1, POLR1B, PRKDC, PTK2B, TRIP13, UBE2C] |
| cell cycle process | 7.52E-18 | regulation of chromosome segregation | 1.33E-04 | [BUB1, CDC6, CDCA5, CENPE, ECT2, KIF2C, MAD2L1, MKI67, NEK2, RACGAP1, TRIP13] |
| cell cycle process | 7.52E-18 | regulation of mitotic cell cycle | 1.63E-04 | [BLM, CDC6, CDCA5, CDK1, CENPE, CENPF, CHEK1, CLSPN, DONSON, DTL, INTS13, KIF14, KNTC1, MAD2L1, MKI67, NEK2, NUSAP1, POLR1B, PRKDC, PTK2B, TOM1L2, TRIP13, UBE2C] |
| cell cycle process | 7.52E-18 | negative regulation of cell cycle process | 3.40E-04 | [BLM, BRCA1, CHEK1, CLSPN, DEPDC1B, DONSON, DTL, FZD3, HSP90AB1, MAD2L1, NEK2, POLR1B, PRKDC, TMEM67, TOM1L2, TRIP13] |
| cell cycle process | 7.52E-18 | G2/M transition of mitotic cell cycle | 1.61E-03 | [BLM, BRIP1, CCNA2, CDC25C, CDK1, CENPF, CLSPN, DONSON, DTL, KIF14] |
| cell cycle process | 7.52E-18 | positive regulation of cell cycle | 2.12E-03 | [ATAD5, BRCA1, CDC6, CDCA5, CDK1, CHEK1, DTL, ECT2, EZH2, FZD3, HSP90AB1, KIF14, KIF23, MAD2L1, NUSAP1, PTK2B, RACGAP1, UBE2C] |
| cell cycle process | 7.52E-18 | cell cycle checkpoint | 2.26E-03 | [BLM, BRCA1, CHEK1, CLSPN, DEPDC1B, DNA2, DONSON, DTL, KNTC1, MAD2L1, POLR1B, PRKDC, TRIP13] |
| cell cycle process | 7.52E-18 | G2 DNA damage checkpoint | 2.60E-03 | [BLM, BRCA1, CHEK1, CLSPN, DEPDC1B, DONSON, DTL] |
| cell cycle process | 7.52E-18 | attachment of spindle microtubules to kinetochore | 3.51E-03 | [CENPE, ECT2, KIF2C, NEK2, RACGAP1, SGO1] |
| cell cycle process | 7.52E-18 | negative regulation of cell cycle phase transition | 6.13E-03 | [BLM, BRCA1, CHEK1, CLSPN, DEPDC1B, DONSON, DTL, MAD2L1, POLR1B, PRKDC, TRIP13] |
| cell cycle process | 7.52E-18 | regulation of mitotic nuclear division | 7.66E-03 | [CDC6, CDCA5, CENPE, CHEK1, MAD2L1, MKI67, NEK2, NUSAP1, TOM1L2, TRIP13, UBE2C] |
| cell cycle process | 7.52E-18 | regulation of nuclear division | 1.09E-02 | [CDC6, CDCA5, CENPE, CHEK1, MAD2L1, MKI67, NEK2, NUSAP1, TOM1L2, TRIP13, UBE2C] |
| cell cycle process | 7.52E-18 | positive regulation of cell cycle process | 1.15E-02 | [ATAD5, BRCA1, CDC6, CDCA5, CDK1, DTL, ECT2, EZH2, KIF14, KIF23, MAD2L1, NUSAP1, RACGAP1, UBE2C] |
| cell cycle process | 7.52E-18 | negative regulation of cell cycle G2/M phase transition | 1.29E-02 | [BLM, BRCA1, CHEK1, CLSPN, DEPDC1B, DONSON, DTL] |
| cell cycle process | 7.52E-18 | negative regulation of cell cycle | 2.93E-02 | [BLM, BRCA1, CDKN3, CHEK1, CLSPN, DEPDC1B, DNA2, DONSON, DTL, FZD3, HSP90AB1, KNTC1, MAD2L1, NEK2, POLR1B, PRKDC, TMEM67, TOM1L2, TRIP13] |
| cell cycle process | 7.52E-18 | regulation of G2/M transition of mitotic cell cycle | 2.94E-02 | [BLM, CDK1, CENPF, CLSPN, DONSON, DTL, KIF14] |
| cell cycle process | 7.52E-18 | DNA damage checkpoint | 3.98E-02 | [BLM, BRCA1, CHEK1, CLSPN, DEPDC1B, DONSON, DTL, POLR1B, PRKDC] |
| DNA repair, cell cycle process | 7.52E-18 | double-strand break repair | 5.75E-07 | [AUNIP, BLM, BRCA1, BRCA2, CDCA5, CHEK1, DEPDC1B, DNA2, EXOSC8, FANCD2, FIGNL1, MMS22L, PARPBP, POLQ, POLR1B, PRKDC, RAD51AP1, RAD54B, XRCC2] |
| DNA repair, cell cycle process | 7.52E-18 | DNA integrity checkpoint | 1.06E-02 | [BLM, BRCA1, CHEK1, CLSPN, DEPDC1B, DNA2, DONSON, DTL, POLR1B, PRKDC] |
| DNA metabolic process | 1.60E-11 | DNA replication | 5.72E-03 | [ATAD5, BLM, BRCA2, DNA2, DONSON, DSCC1, FANCD2, GTPBP4, MMS22L, POLR1B, RFC3, RFC4] |
| DNA metabolic process | 1.60E-11 | DNA-dependent DNA replication | 1.41E-02 | [BLM, BRCA2, DNA2, DONSON, DSCC1, FANCD2, MMS22L, RFC3, RFC4] |
| DNA metabolic process | 1.60E-11 | regulation of DNA replication | 1.57E-02 | [ATAD5, BLM, DNA2, DONSON, DSCC1, GTPBP4, RFC3, RFC4] |
| mitotic cytokinesis | 1.53E-08 | mitotic cytokinesis | 5.27E-06 | [ANLN, BRCA2, CENPA, CEP55, CKAP2, ECT2, FANCD2, KIF23, KIF4A, NUSAP1, RACGAP1] |
| mitotic cytokinesis | 1.53E-08 | microtubule cytoskeleton organization | 1.33E-05 | [ASPM, AUNIP, AURKA, BRCA2, CENPA, CENPE, CHEK1, FANCD2, INTS13, KIF18A, KIF23, KIF2C, KIF4A, NEK2, NUSAP1, RACGAP1, SGO1, SKA1, SKA3, SLC25A23, SPC25, STIL, TMEM67, TPX2, XRCC2] |
| mitotic cytokinesis | 1.53E-08 | cytoskeleton-dependent cytokinesis | 2.28E-05 | [ANLN, BRCA2, CENPA, CEP55, CKAP2, ECT2, FANCD2, KIF23, KIF4A, NUSAP1, RACGAP1] |
| mitotic cytokinesis | 1.53E-08 | cytokinesis | 1.97E-03 | [ANLN, BRCA2, CDC6, CENPA, CEP55, CKAP2, ECT2, FANCD2, KIF14, KIF23, KIF4A, NUSAP1, RACGAP1] |
| mitotic cytokinesis | 1.53E-08 | spindle organization | 1.02E-02 | [ASPM, AUNIP, AURKA, CENPE, INTS13, KIF23, KIF4A, RACGAP1, SPC25, STIL, TPX2] |
| mitotic cytokinesis | 1.53E-08 | microtubule cytoskeleton organization involved in mitosis | 1.45E-02 | [CENPA, CENPE, INTS13, KIF23, KIF4A, NUSAP1, RACGAP1, SPC25, STIL, TPX2] |
| DNA metabolic process, DNA repair | 2.69E-06 | DNA metabolic process | 4.78E-10 | [ATAD5, AUNIP, BLM, BRCA1, BRCA2, BRIP1, CCT6A, CDCA5, CDKN3, CHEK1, DEPDC1B, DKC1, DNA2, DONSON, DSCC1, DTL, ERCC6L, EXO1, EXOSC8, FANCD2, FIGNL1, GNL3, GTPBP4, HSP90AB1, HSPD1, MMS22L, NAT10, NEIL3, NEK2, PARPBP, POLQ, POLR1B, PRKDC, PTK2B, RAD51AP1, RAD54B, RFC3, RFC4, TOP2A, UBE2T, XRCC2] |
| DNA metabolic process, DNA repair | 2.69E-06 | regulation of DNA metabolic process | 3.03E-06 | [ATAD5, AUNIP, BLM, BRCA1, CCT6A, CDKN3, CHEK1, DEPDC1B, DKC1, DNA2, DONSON, DSCC1, FIGNL1, GNL3, GTPBP4, HSP90AB1, NAT10, NEK2, PARPBP, POLQ, POLR1B, PTK2B, RAD51AP1, RFC3, RFC4] |
| DNA metabolic process, DNA repair | 2.69E-06 | positive regulation of DNA metabolic process | 1.04E-03 | [ATAD5, BLM, BRCA1, CCT6A, CDKN3, DEPDC1B, DKC1, DNA2, DSCC1, GNL3, HSP90AB1, NEK2, PTK2B, RFC3, RFC4] |
| DNA metabolic process, DNA repair | 2.69E-06 | regulation of DNA repair | 5.99E-03 | [AUNIP, BRCA1, CDKN3, CHEK1, DEPDC1B, FIGNL1, PARPBP, POLQ, POLR1B, RAD51AP1] |
| DNA repair | 2.69E-06 | DNA repair | 6.25E-08 | [AUNIP, BLM, BRCA1, BRCA2, BRIP1, CDCA5, CDKN3, CHEK1, DEPDC1B, DNA2, DTL, ERCC6L, EXO1, EXOSC8, FANCD2, FIGNL1, MMS22L, NEIL3, PARPBP, POLQ, POLR1B, PRKDC, RAD51AP1, RAD54B, UBE2T, XRCC2] |
| DNA repair | 2.69E-06 | DNA recombination | 2.55E-06 | [AUNIP, BLM, BRCA1, BRCA2, CHEK1, EXO1, FANCD2, FIGNL1, HSPD1, MMS22L, PARPBP, POLQ, POLR1B, RAD51AP1, RAD54B, XRCC2] |
| DNA repair | 2.69E-06 | double-strand break repair via homologous recombination | 1.05E-05 | [AUNIP, BRCA1, BRCA2, CHEK1, FANCD2, FIGNL1, MMS22L, PARPBP, POLQ, POLR1B, RAD51AP1, RAD54B, XRCC2] |
| DNA repair | 2.69E-06 | recombinational repair | 1.19E-05 | [AUNIP, BRCA1, BRCA2, CHEK1, FANCD2, FIGNL1, MMS22L, PARPBP, POLQ, POLR1B, RAD51AP1, RAD54B, XRCC2] |
| DNA repair | 2.69E-06 | cellular response to DNA damage stimulus | 1.38E-05 | [AUNIP, BLM, BRCA1, BRCA2, BRIP1, CDCA5, CDKN3, CHEK1, CLSPN, DDIAS, DEPDC1B, DNA2, DONSON, DTL, E2F7, ERCC6L, EXO1, EXOSC8, FANCD2, FIGNL1, MCM10, MMS22L, NEIL3, PARPBP, PMAIP1, POLQ, POLR1B, PRKDC, RAD51AP1, RAD54B, TOP2A, UBE2T, XRCC2] |
| DNA repair | 2.69E-06 | regulation of double-strand break repair via homologous recombination | 1.28E-02 | [CHEK1, FIGNL1, PARPBP, POLQ, POLR1B, RAD51AP1] |
| DNA repair | 2.69E-06 | cellular response to stress | 1.48E-02 | [AUNIP, BLM, BRCA1, BRCA2, BRIP1, CDC25C, CDCA5, CDKN3, CHEK1, CLSPN, DDIAS, DEPDC1B, DNA2, DONSON, DTL, E2F7, ECT2, EEF1E1, ERCC6L, EXO1, EXOSC8, FANCD2, FIGNL1, HILPDA, MCM10, MMS22L, NEIL3, PARPBP, PDK2, PINK1, PMAIP1, POLQ, POLR1B, PRKDC, PSAT1, PTK2B, RAD51AP1, RAD54B, RBL1, SERPINF2, SUV39H2, TMEM67, TOP2A, UBE2T, XRCC2] |
| DNA repair | 2.69E-06 | regulation of DNA recombination | 1.66E-02 | [BLM, CHEK1, FIGNL1, PARPBP, POLQ, POLR1B, RAD51AP1] |
| protein localization to chromosome | 3.45E-05 | protein localization to chromosome | 1.03E-03 | [BRCA2, BUB1B, CCT6A, CDCA5, CDK1, CENPA, DKC1, FANCD2, GNL3, MTBP, POLR1B] |
| chromosome condensation | 4.78E-04 | chromosome condensation | 1.43E-02 | [CDCA5, NCAPG, NCAPH, NUSAP1, TOP2A] |
| chromosome condensation | 4.78E-04 | mitotic chromosome condensation | 4.47E-02 | [CDCA5, NCAPG, NCAPH, NUSAP1] |
| rRNA processing | 1.73E-03 | rRNA processing | 3.18E-03 | [DKC1, HEATR1, LYAR, MPP6, TTK, WDR12, WDR43, WDR75] |
| rRNA processing | 1.73E-03 | ribosome biogenesis | 1.82E-02 | [DDX31, DKC1, HEATR1, LYAR, MPP6, TTK, WDR12, WDR43, WDR75] |
| rRNA processing | 1.73E-03 | ncRNA metabolic process | 4.05E-02 | [BRCA1, DKC1, GARS, HEATR1, IARS, LYAR, MPP6, POP1, PUS7, TTK, WDR12, WDR43, WDR75, ZC3H8] |

**Table S6.** ClueGO-CluePedia functional analysis of green module in LSCRC from cohort 2.

| GO Functional Groups | Group *P* Value | GO Term | Term *P* Value | Associated Genes Found |
| --- | --- | --- | --- | --- |
| regulation of cardiac muscle cell action potential | 4.46E-03 | regulation of cardiac muscle cell action potential | 3.83E-02 | [AKAP9, APC, DSC2, SLMAP] |
| regulation of cardiac muscle cell action potential | 4.46E-03 | regulation of membrane depolarization | 4.37E-02 | [CAMK2D, NCOA1, NEDD4L, SLMAP] |
| regulation of cardiac muscle cell action potential | 4.46E-03 | regulation of membrane potential | 4.60E-02 | [AKAP7, AKAP9, APC, CAMK2D, DSC2, NCOA1, NEDD4L, PYCR1, SLMAP, SYTL2] |
| activation of immune response | 4.81E-03 | activation of immune response | 3.99E-02 | [BCL10, BIRC3, DDX60, DENND1B, EHHADH, ERBIN, IRAK1, PDE4D, PJA2, RIOK3, RNF125, SYTL2, TLR3, TP53INP1] |
| activation of immune response | 4.81E-03 | pattern recognition receptor signaling pathway | 4.30E-02 | [BIRC3, DDX60, EHHADH, ERBIN, IRAK1, PJA2, RIOK3, RNF125, TLR3] |
| activation of immune response | 4.81E-03 | innate immune response-activating signal transduction | 4.30E-02 | [BIRC3, DDX60, EHHADH, ERBIN, IRAK1, PJA2, RIOK3, RNF125, TLR3] |
| activation of immune response | 4.81E-03 | positive regulation of immune response | 4.33E-02 | [B2M, BCL10, BIRC3, DDX60, DENND1B, EHHADH, ERBIN, IRAK1, PDE4D, PJA2, RIOK3, RNF125, SLC27A5, SYTL2, TLR3, TP53INP1, ZP3] |
| activation of immune response | 4.81E-03 | positive regulation of innate immune response | 4.50E-02 | [BIRC3, DDX60, EHHADH, ERBIN, IRAK1, PJA2, RIOK3, RNF125, SLC27A5, TLR3, TP53INP1] |
| activation of immune response | 4.81E-03 | positive regulation of defense response | 4.53E-02 | [BIRC3, DDX60, EHHADH, ERBIN, F12, IRAK1, PJA2, RIOK3, RNF125, SLC27A5, TLR3, TP53INP1] |
| activation of immune response | 4.81E-03 | activation of innate immune response | 4.95E-02 | [BIRC3, DDX60, EHHADH, ERBIN, IRAK1, PJA2, RIOK3, RNF125, TLR3, TP53INP1] |
| regulation of NIK/NF-kappaB signaling | 5.76E-03 | regulation of NIK/NF-kappaB signaling | 4.32E-02 | [CPNE1, IRAK1, PDCD4, PPM1A, RAP1A, TCIM, TLR3] |
| regulation of NIK/NF-kappaB signaling | 5.76E-03 | NIK/NF-kappaB signaling | 4.53E-02 | [CPNE1, IRAK1, PDCD4, PPM1A, RAP1A, TCIM, TLR3] |
| centriole assembly | 6.60E-03 | centriole assembly | 4.44E-02 | [CCDC78, KAT2A, KAT2B, TP53INP1, VPS4B] |
| centriole assembly | 6.60E-03 | regulation of centriole replication | 4.68E-02 | [KAT2A, KAT2B, TP53INP1, VPS4B] |
| neutral amino acid transport | 7.13E-03 | neutral amino acid transport | 4.20E-02 | [DDX39A, SLC1A1, SLC1A5, SLC43A1] |
| fatty acid beta-oxidation using acyl-CoA dehydrogenase | 8.72E-03 | fatty acid beta-oxidation using acyl-CoA dehydrogenase | 4.11E-02 | [ACADM, ETFBKMT, ETFDH] |
| necrotic cell death | 9.79E-03 | necrotic cell death | 4.20E-02 | [BIRC3, FAS, FASN, NPM3, TLR3] |

1. **Supplementary Methods**

**3.1 Clinical specimens**

For detecting of protein in cohort 1, the patients (*N* = 207) with primary colorectal cancer from the Southeast Swedish Health Care region, including hospitals in Linköping, Norrköping, Motala, Jönköping, Kalmar, Oskarshamn, Västervik, Eksjö, and Värnamo were analyzed. Their corresponding distant normal mucosa (*N* = 39), and metastases in the regional lymph nodes (*N* = 21) were also included in the present study. The clinicopathological characteristics, including age, gender, tumor subsites (RSCC and LSCRC), growth pattern (expansive and infiltrative) differentiation were obtained from surgical and pathological records, TNM staging was performed according to the American Joint Committee on Cancer (AJCC). Information on vital status and cause of death was obtained from the Swedish Cause of Death Registry up until 31 December 2013. The required informed consents were given to all participants. The detailed parameters were summarized in Supplementary Table S1.

For detecting of mRNA level in cohort 2, the level-3 data of RNA-seq and clinicopathology of TCGA colorectal samples (COREAD) were obtained from UCSC Xena (https://xenabrowser.net/hub/). The primary colorectal cancer tissue (*N* = 596) and the normal colorectal mucosa(*N* = 51) were included, after excluding cases without clinical survival data. The detailed parameters were summarized in Supplementary Table S2.

**3.2 Immunohistochemistry and staining evaluation**

The five-micrometer paraffin-embedded tissue sections were deparaffinized in xylene and rehydrated with a series of gradient ethanol to water. The sections were heated to boiling point in citrate buffer (pH 6.0) for 30 min to unmask antigen, followed by a washing in phosphate-buffered saline (PBS). Endogenous peroxidase activity was blocked with 3% H_2_O_2_ in methanol followed by washing three times in PBS. The sections were incubated with protein block (Dako, Carpinteria, CA) for 10 min and then incubated with mouse monoclonal MRE11 antibody (Abcam, Cambridge, UK) at 4°C overnight. After that, the sections were washed in PBS and then incubated with goat anti-mouse secondary antibody (Dako, Carpinteria, CA) at room temperature for 25 min. Next, the sections were subjected to 3,3’-diaminobenzidine tetrahydrochloride for 8 min and then counterstained with hematoxylin. Negative and positive controls were added in each staining run. All slides were scored by two independent investigators. Images of were captured with an Aperio CS2 slide scanner system (Leica Biosystems) using a 40x magnification. The percentage of MRE11 positive tumor cells was classified as high expression and low expression based on setting 75% as a cut-off point, regardless of the expression intensity.

**3.3 The evaluation of tumor-infiltrating inflammatory cells,**

The evaluation of tumor-infiltrating inflammatory cells (TIICs) followed previous study(1). Briefly, TIICs were identified as small mononuclear cells in the stroma of the tumor and TIICs in the margins of the sections were not included in order to avoid artifacts. The distributions of the infiltration were classified into two groups by localization: (a) those presented along with the invasive margin of the tumor; (b) those distributed in entire tumor. The degree of infiltration was classified as absent, sparse, moderate, and intense according to the density of inflammatory cells. Since the distributions of TIICs were often heterogeneous, the entire sections were examined to assess tumor areas including high and low TIICs. But, only marginal infiltration of TIICs was included in present study.

**3.4 Microsatellite testing and analysis**

The microsatellite status (MSS; MSI) of all samples from cohort 1 was identified by PCR based assays following previous study (2).

**3.5 Construction of weighted gene co-expression network**

The R package WGNCA was performed for co-expression network constructions (3). 5086 differentially expressed genes (DEGs) were included in the network. First, one outlier samples were removed from the subsequent analysis (Supplementary Fig. S3A). A power of 3 was selected, in accordance with networks’ connectivity distributions approximate the power law, indicating the network possesses scale-free topology. The topological overlap distance calculated from the adjacency matrix was then clustered with the average linkage hierarchical clustering. To obtain moderately large and distinct modules, we set the minimum module size to 40 genes and the minimum height for merging modules at 0.15.

Next, we determined correlations among gene expression modules and clinical traits. The MSI status, histological type, lymphatic invasion, venous invasion, and MRE11 expression in left-sided and right-sided CRC were chosen as clinical traits. In addition, the association of gene significance (GS) and module membership (MM) was also assessed how close significance of gene expression is to the module. The data and scripts can be found on the GitHub page as the link: https://github.com/xuntian2005/MRE11_WGCNA.

**3.6 ClueGO-CluePedia functional analysis**

To investigate the biological role of the genes of the module identified, we used a Cytoscape plugin app, ClueGO and CluePedia, which presents enriched pathways within a network, interconnected based on kappa score (4). In the present analysis, GO evidence was limited in all experimental model. Terms found in the 3-8 GO interval, with at least 3 genes from the initial list representing minimum 3% were selected. An adjusted *P* value of <0.01 or <0.05 and a kappa coefficient of 0.4 were considered as threshold values.

**3.7 Statistical analyses**

Data analysis was performed by using SPSS 22.0 software package (SPSS, Inc., IL, USA). The χ^2^ test method were used to determine the difference in expression of the proteins among normal mucosa, primary tumor, and metastasis, as well as the relationship of the proteins’ expression in primary CRC with clinicopathological features in cohort 1. Two-tailed, student *t-test or* one-way ANOVA was performed to compare numerical values in cohort 2. To assess MRE11 as a prognostic biomarker, patient samples analyzed were dichotomized into two groups designed as high- and low- groups, based on an MRE11 cutoff value (8.7368) in cohort2. Survival curves were estimated using the Kaplan-Meier with the log-rank test. Univariate and multivariate Cox tests were performed to assess the relative contribution of the risk group when assessed alone or after adjusting for clinical variables. Kaplan–Meier curves of OS of the patients in cohort1 and cohort2 were generated by GraphPad Prism 6.0. The *p* < 0.05 was considered statistically significance. For DEGs screening in cohort 2, R package “limma” was applied to select the DEGs by comparing 596 CRC samples and 51 normal colorectal mucosa samples. The cut-off criteria for screening DEGs was the false discovery rate (FDR) < 0.05 and |log2(fold change) | >1.

**Supplementary references**

1. Gao JF, Arbman G, Wadhra TI, Zhang H, Sun XF. Relationships of tumor inflammatory infiltration and necrosis with microsatellite instability in colorectal cancers. *World J Gastroenterol* (2005) 11(14):2179-83. PubMed PMID: 15810089; PubMed Central PMCID: PMC4305792.

2. Gao J, Zhang H, Arbman G, Sun XF. RAD50/MRE11/NBS1 proteins in relation to tumour development and prognosis in patients with microsatellite stable colorectal cancer. *Histol Histopathol* (2008) 23(12):1495-502. doi: 10.14670/HH-23.1495. PubMed PMID: 18830935.

3. Langfelder P, Horvath S. WGCNA: an R package for weighted correlation network analysis. *Bmc Bioinformatics* (2008) 9:559. doi: 10.1186/1471-2105-9-559. PubMed PMID: 19114008; PubMed Central PMCID: PMC2631488.

4. Mlecnik B, Bindea G, Angell HK, Maby P, Angelova M, Tougeron D, et al. Integrative Analyses of Colorectal Cancer Show Immunoscore Is a Stronger Predictor of Patient Survival Than Microsatellite Instability. *Immunity* (2016) 44(3):698-711. doi: 10.1016/j.immuni.2016.02.025. PubMed PMID: 26982367.
